# The intersection of biological sex and gender in adverse events following seasonal influenza vaccination in older adults

**DOI:** 10.1186/s12979-023-00367-3

**Published:** 2023-08-29

**Authors:** Janna R. Shapiro, Kumba Seddu, Han-Sol Park, John S. Lee, Patrick S. Creisher, Anna Yin, Patrick Shea, Helen Kuo, Huifen Li, Engle Abrams, Sean X. Leng, Rosemary Morgan, Sabra L. Klein

**Affiliations:** 1 W. Harry Feinstone Department of Molecular Microbiology and Immunology, Johns Hopkins Bloomberg School of Public Health, 615 N. Wolfe Street, Baltimore, MD 21205 USA; 2 Division of Geriatric Medicine and Gerontology, Department of Medicine, Johns Hopkins University School of Medicine, Baltimore, MD USA; 3 Department of International Health, Johns Hopkins Bloomberg School of Public Health, Baltimore, MD USA

**Keywords:** Sex, Gender, BSRI, Adverse events, Influenza, Older adults, Estradiol, IL-6

## Abstract

**Background:**

Women/females report more adverse events (AE) following immunization than men/males for many vaccines, including the influenza and COVID-19 vaccines. This discrepancy is often dismissed as a reporting bias, yet the relative contributions of biological sex and gender are poorly understood. We investigated the roles of sex and gender in the rate of AE following administration of the high-dose seasonal influenza vaccine to older adults (≥ 75 years) using an AE questionnaire administered 5–8 days post-vaccination. Participant sex (male or female) was determined by self-report and a gender score questionnaire was used to assign participants to one of four gender categories (feminine, masculine, androgynous, or undifferentiated). Sex steroid hormones and inflammatory cytokines were measured in plasma samples collected prior to vaccination to generate hypotheses as to the biological mechanism underpinning the AE reported.

**Results:**

A total of 423 vaccines were administered to 173 participants over four influenza seasons (2019-22) and gender data were available for 339 of these vaccinations (2020-22). At least one AE was reported following 105 vaccinations (25%), by 23 males and 82 females. The majority of AE occurred at the site of injection, were mild, and transient. The odds of experiencing an AE were 3-fold greater in females than males and decreased with age to a greater extent in females than males. The effects of gender, however, were not statistically significant, supporting a central role of biological sex in the occurrence of AE. In males, estradiol was significantly associated with IL-6 and with the probability of experiencing an AE. Both associations were absent in females, suggesting a sex-specific effect of estradiol on the occurrence of AE that supports the finding of a biological sex difference.

**Conclusions:**

These data support a larger role for biological sex than for gender in the occurrence of AE following influenza vaccination in older adults and provide an initial investigation of hormonal mechanisms that may mediate this sex difference. This study highlights the complexities of measuring gender and the importance of assessing AE separately for males and females to better understand how vaccination strategies can be tailored to different subsets of the population.

**Supplementary Information:**

The online version contains supplementary material available at 10.1186/s12979-023-00367-3.

## Background

For many vaccines, including the seasonal influenza vaccine, it has consistently been reported that females/women experience higher rates of adverse events (AE) than males/men [1–8]. The literature provides evidence through which both sex, the biological differences between males and females based on sex chromosome complement and sex steroids, and gender, the socio-cultural differences between men and women, may contribute to this effect [8, 9]. The relative contribution of these two constructs remains unclear, and without a known biological underpinning, many dismiss this effect as solely a gendered reporting bias [2, 10]. While femininity has consistently been associated with lower pain tolerance [11, 12] and more socially acceptable expressions of pain [2, 10, 13], there are important anatomical [2, 14–16] and immunological differences between males and females that may contribute to sex differences in the physiological pathways that lead to the occurrence of AE.

Immunologically, AE are physical manifestations of the inflammatory response that occurs when vaccine antigens stimulate the release of vasodilators and pyrogenic cytokines (i.e., IL-6, TNFα), recruiting cells to the site of injection and causing redness, swelling, and pain [17]. When these inflammatory mediators spill into the bloodstream, they initiate a response in the central nervous system that can cause systemic reactions, such as fever, headache, and myalgia [17]. Both older and younger females release higher levels of inflammatory cytokines in response to influenza vaccination than males, a process that may be mediated by sex steroid hormones, such as estradiol and testosterone, suggesting a biological mechanism through which AE are more likely to occur in females [18–23].

Understanding the relative contributions of both sex and gender in the occurrence and reporting of AE is necessary to mitigate the significant impact of perceptions of vaccine safety on vaccine acceptance [24–26]. Dismissing women’s experiences as solely due to lower pain threshold or higher willingness to report may obscure the biological mechanisms causing sex differences in AE and prohibit a deeper understanding of how vaccine strategies can be tailored to different subsets of the population. Here, we performed active surveillance for AE following influenza vaccination in a cohort of adults above 75 years of age and measured the associations of both sex and gender with AE reporting. To measure gender, we used a short-form Bem Sex Role Inventory (BSRI) to calculate femininity and masculinity scores and to assign participants to one of four gender categories – feminine, masculine, androgynous, or undifferentiated. We further assessed the roles of sex steroid hormones and inflammatory cytokines to generate hypotheses as to the sex-specific mechanisms that mediate AEs in older adults.

## Results

### Participant characteristics

**Fig. 1:**
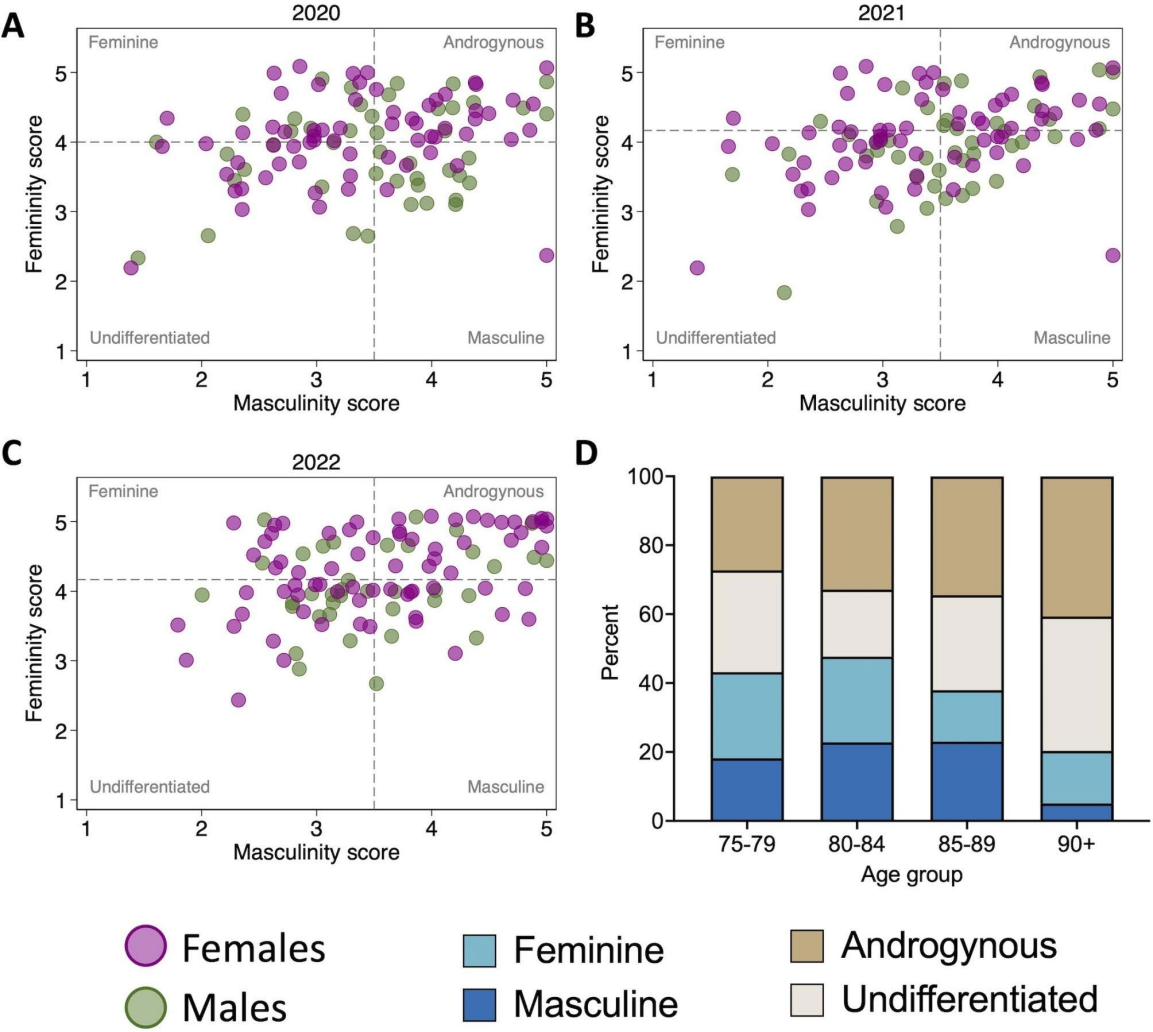
Gender dynamics. Gender was measured in the 2020-21 (**A**), 2021-22 (**B**), and 2022–2023 (**C**) influenza seasons using a modified version of the short-form Bem Sex Role Inventory. Based on responses to questions about six instrumental/masculine (I/M) traits (leadership abilities, strong personality, acts as leader, dominant, makes decisions easily, defends own beliefs) and six expressive/feminine (E/F) traits (warm, gentle, affectionate, sympathetic, sensitive to other’s needs, tender), mean femininity and masculinity scores were calculated and compared to the population median to assign participants into one of four gender categories. Dashed lines represent the median femininity and masculinity scores for each year (**A-C**). The distribution of the four gender categories by age group is shown in **D**

Over four influenza seasons from 2019 to 2022, 173 participants received a total of 423 influenza vaccinations (Table 1). The study population was predominantly female (61%) and the mean age at vaccination was 85 years for males and 84 years for females. Gender-associated factors were measured in the 2020–2022 seasons, resulting in a ‘gender subset’ comprised of data for 339 vaccinations administered to 162 individuals. Overall, androgyny was the most common gender category, and femininity scores were higher than masculinity scores for both males and females (Table 1**and** Fig. 1A-C). The proportion of participants who were classified as either feminine or masculine decreased markedly with age, and the prevalence of androgynous and undifferentiated people increased accordingly (Fig. 1D).

**Table 1:** Participant characteristics

	All	Male	Female
**Full data set (2019-22)**			
Individuals - N (%)	173 (100)	67 (39)	106 (61)
Vaccination events - N (%)	423 (100)	166 (39)	257 (61)
Age at vaccination			
Mean (min-max)	85 (75–99)	85 (76–99)	84 (75–99)
Median (IQR)	84 (81–88)	84 (82–88)	83 (81–88)
**Gender subset (2020-22)**			
Individuals - N (%)	162 (100)	62 (38)	100 (62)
Vaccination events - N (%)	339 (100)	127 (37)	212 (63)
Age at vaccination			
Mean (min-max)	85 (75–99)	85 (76–99)	84 (75–99)
Median (IQR)	84 (81–88)	84 (82–88)	83 (81–88)
Gender category - N (%)			
Masculine	65 (19)	37 (29)	28 (13)
Feminine	70 (21)	21 (17)	49 (23)
Androgynous	115 (34)	35 (28)	80 (38)
Undifferentiated	89 (26)	34 (27)	55 (26)
Masculinity score			
Mean (min-max)	3.5 (1.3–5.0)	3.5 (1.5–5.0)	3.5 (1.3–5.0)
Median (IQR)	3.5 (3.0–4.2)	3.5 (3.0–4.0)	3.5 (2.8–4.2)
Femininity score			
Mean (min-max)	4.1 (1.8–5.0)	4.0 (1.8–5.0)	4.2 (2.2–5.0)
Median (IQR)	4.2 (3.7–4.7)	4.0 (3.5–4.5)	4.2 (3.8–4.7)
**Serology subset (2020)**			
Vaccination events - N (%)	112 (100)	46 (41)	66 (59)
Age at vaccination			
Mean (min-max)	85 (75–98)	85 (76–98)	84 (75–98)
Median (IQR)	83 (81–88)	84 (82–87)	83 (81–88)

### Adverse events were mild and transient

**Fig. 2:**
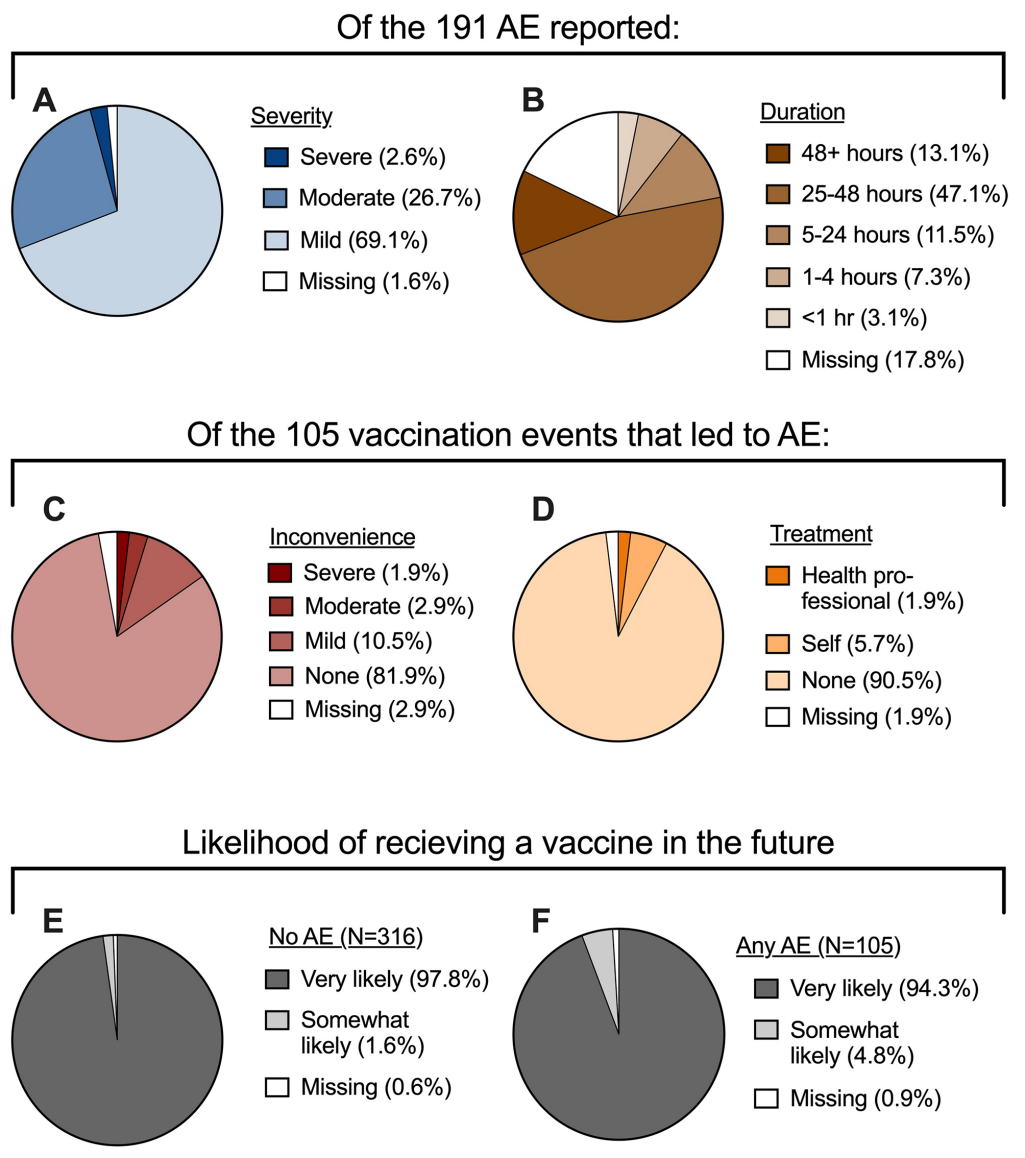
Severity and impact of adverse events (AE). For each AE reported, participants were asked to rate the severity (**A**) and indicate the duration of the event (**B**). Each participant who experienced at least one AE following vaccination was then asked to rate the level of inconvenience that their AE(s) posed for completing activities of daily living (**C**) and what type of treatment, if any, was sought for the AE (**D**). Participant willingness to receive a vaccination in the future was then stratified by AE status (**E-F**).

Over the four study years, 105 vaccination events led to at least one AE, with a total of 191 AE reported. The majority of AE (69%) were rated as mild by the participant (Fig. 2A). Fifty-one (27%) and five (3%) were reported as moderate or severe, respectively. Furthermore, of the 157 AE for which duration data was available, 132 (84%) lasted less than 48 h (Fig. 2B). For the 105 vaccination events that led to at least one AE, participants were asked how the AE affected their ability to perform activities of daily living and whether they sought any treatment. Most participants (82%) reported that their AE had no effect on their ability to perform activities of daily living, with only two female participants reporting that they were severely inconvenienced (Fig. 2C). Similarly, 91% of participants did not seek any type of treatment for their AE (Fig. 2D). Six participants (5/6 female) reported self-treatment of the AE, usually in the form of over-the-counter pain medication. Two female participants sought treatment from a healthcare professional. Despite the mild and transient nature of the AEs reported, more participants who reported an AE said they were “somewhat likely” to receive a vaccine in the future as opposed to “very likely” (Fig. 2E-F). Finally, from an immunogenicity perspective, there were no significant differences in microneutralization outcomes (geometric mean titers, geometric mean fold rise from day 0 to day 28, or seroconversion) between participants who reported an AE and those who did not, suggesting no benefit of AE on vaccine-induced antibody responses (Supplemental Table 1). Overall, these data suggest that AE did not have a significant impact on the participant’s well-being or antibody response but may affect willingness to be vaccinated in the future.

### Biological sex and age impact the likelihood of reporting AE

**Fig. 3:**
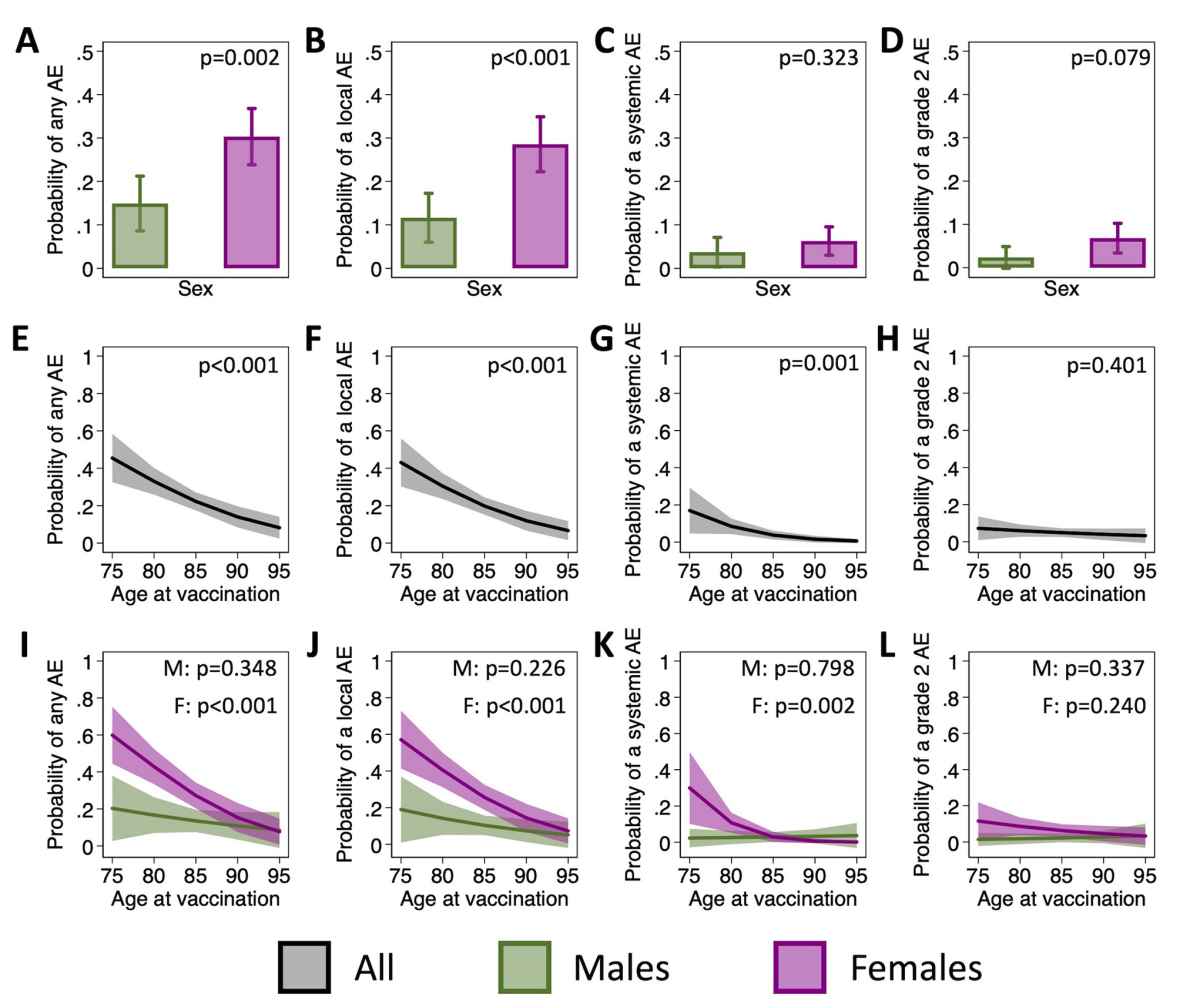
Impact of sex, age, and their interaction on adverse event (AE) reporting. The estimated probabilities of reporting of any adverse event (AE) (**A**), any local AE (**B**), and any systemic AE (**C**) are shown for males and females. Probabilities were estimated using mixed effects regression models to account for repeat measurements on individuals over the four influenza seasons and control for age at vaccination. Using similar models, but controlling for sex, the change in the probability of reporting an AE with age is shown (**D-F**). Expanded models with an interaction term between age and sex were then used to assess the effect of age separately for males and females (**G-I**).

A greater proportion of females than males reported each solicited AE in each of the four composite outcomes (i.e., any AE, any local AE, any systemic AE, and any grade 2 AE) (Table 2). Accordingly, base univariate models confirmed a significant sex difference in the reporting of any AE (OR = 4.0, p < 0.001) and any local AE (OR = 5.2, p < 0.001) (Supplemental Table 2). The trend towards greater reporting in females held true for systemic AE and grade 2, but the sex difference failed to reach statistical significance (OR = 2.9, p = 0.135 for systemic AE and OR = 3.75, p = 0.062), likely due to the low overall rate of systemic AE. Findings were consistent, although point estimates slightly attenuated, when adjusting for age (Fig. 3A-D & Supplemental Table 2). Together, these data support a role for biological sex in the occurrence of AE following influenza vaccination in older adults.

**Table 2:** Solicited adverse events by sex

	All	Male	Female
**Vaccination events - N**	423	166	257
**Any adverse event – N (%)**	105 (25)	23 (14)	82 (32)
**Any local adverse event – N (%)**	96 (23)	18 (11)	78 (30)
Redness	26 (10)	2 (1)	24 (9)
Swelling	22 (9)	3 (2)	19 (7)
Itching	29 (11)	4 (2)	25 (10)
Warmth	18 (7)	5 (3)	13 (5)
Pain on contact	51 (20)	9 (5)	42 (16)
Continuous pain	17 (7)	3 (2)	14 (5)
**Any systemic adverse event – N (%)**	22 (5)	5 (3)	17 (7)
Fever	1 (0)	0 (0)	1 (0)
Sweating	0 (0)	0 (0)	0 (0)
Chills	2 (1)	0 (0)	2 (1)
Muscle aches	13 (5)	3 (2)	10 (4)
Headaches	5 (2)	1 (1)	4 (2)
Malaise	6 (2)	1 (1)	5 (2)
Insomnia	0 (0)	0 (0)	0 (0)
**Adverse events by grade**			
Grade 1^a^	83 (20)	19 (11)	64 (25)
Grade 2^b^	22 (5)	4 (2)	18 (7)

^a^ Adverse event did not affect activities of daily living and no treatment was sought

^b^ Adverse event impacted activities of daily living or treatment was sought

We next assessed the role of age in the reporting of AE. Both base models and expanded models adjusting for sex revealed that the odds of reporting any AE, a local AE, or a systemic AE decreased significantly with age (Fig. 3E-G & Supplemental Table 2). This trend did not hold true, however, for grade 2 AE. In models that included an interaction term between age and sex, based on *a priori* hypotheses about the sex-specific effects of aging, the effect of aging on the reporting of AE tended to be greater in females than in males (Fig. 3I-K & Supplemental Table 2). For example, the odds of reporting any AE decreased by 17% per year in females (OR = 0.83, p < 0.001) and by 6% per year in males (OR = 0.94, p = 0.348), although the sex difference in the effect of age was not statistically significant. These data demonstrate a greater effect of age on the rate of AE in females than males, and further support a role for biological sex.

### Gender categories and scores do not affect adverse events reporting

**Fig. 4:**
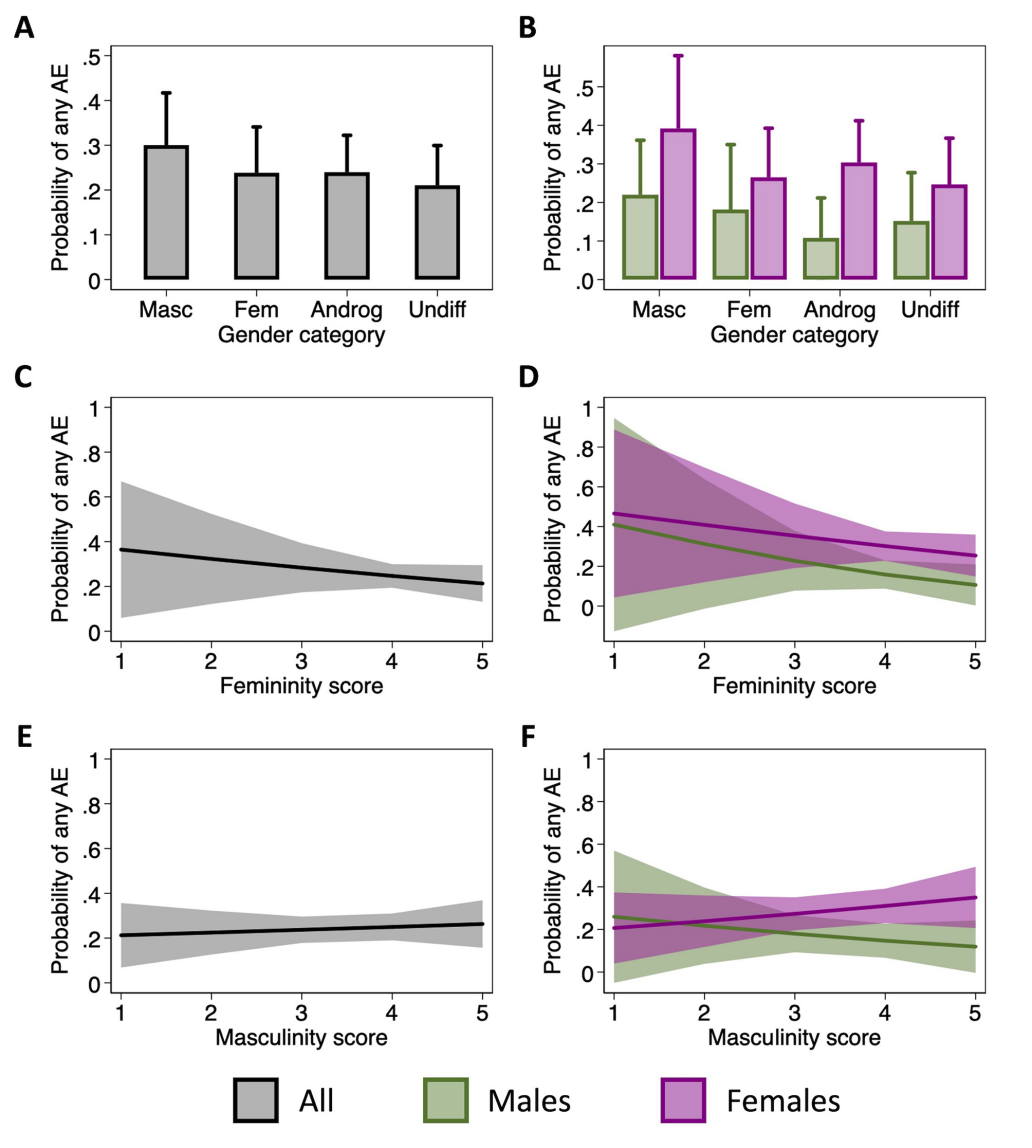
Impact of gender categories and scores on adverse event (AE) reporting. The probability of reporting any AE in each gender category was estimated using mixed effects logistic regression models to account for repeat measurements on participants over three influenza seasons (**A**). The model was then expanded to include an interaction term between sex and gender (**B**). Using similar models, the effect of femininity (**C-D**) and masculinity (**E-F**) scores on the probability of reporting any AE was estimated for the overall population (**C & E**) and separately for males and females (**D & F**). No p-values are shown because no comparisons were statistically significant

To evaluate the role of gender in the reporting of AE, we used the short-form BSRI to calculate femininity and masculinity scores and to assign participants to one of four gender categories – feminine, masculine, androgenous or undifferentiated. Reporting of any AE by gender category and biological sex is described in Supplemental Table 3. Overall, and within each sex, a greater percentage of those who were classified as masculine reported an AE than in the other categories. In mixed effects logistic regression analyses, there were no significant differences in the odds of reporting an AE between gender groups overall or among either males or females (Fig. 4A-B & Supplemental Table 3). Within each gender category, the females tended to have a higher probability of reporting AE than the males. Similarly, neither the femininity nor the masculinity scores influenced the odds of reporting for either males or females (Fig. 4C-F & Supplemental Table 3). Taken together, these data suggest that gender, as measured by the BSRI, does not have a significant impact on the reporting of AE following immunization among older adults.

### Number of AE per vaccination event was associated with sex and age, but not gender

**Fig. 5:**
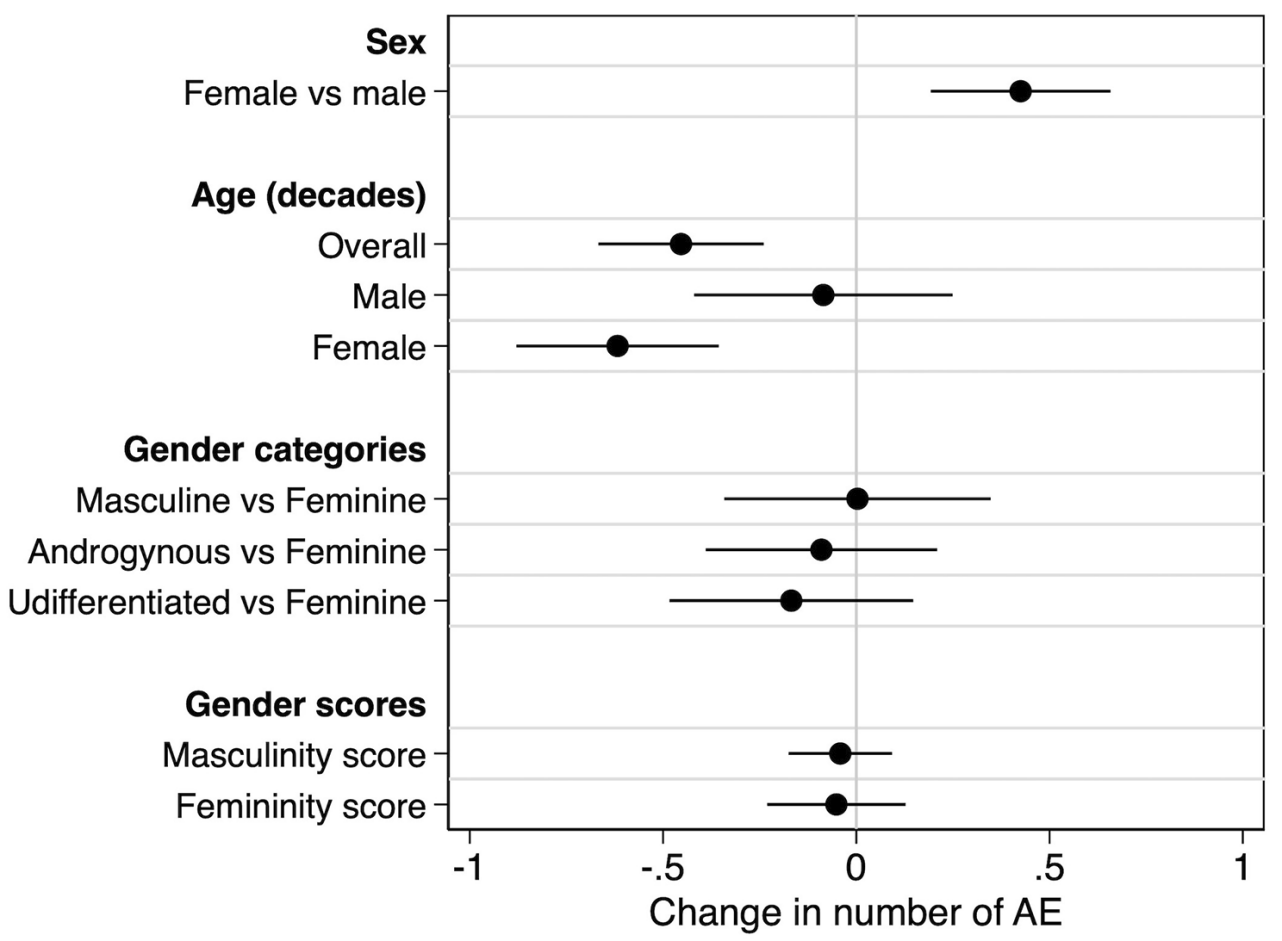
Impact of sex, age, and gender on the number of adverse events (AE) reported per participant. Univariate mixed effects linear regression models were used to estimate the change in number of adverse events (AE) reported per participant between males and female, for a decade increase in age, between gender categories (with feminine used as the reference category), and for each unit increase in gender scores

Analyses investigating the roles of sex, age, and gender were repeated using the number of AE reported per vaccination event as an alternate outcome. Consistent with the sex differences described above, the number of AE per vaccination event was higher in females than males (p < 0.001; Fig. 5). Similar trends also were observed for age, where the number of AE decreased by 0.5 per decade in the overall population (p < 0.001). In sex-disaggregated analyses, this trend was significant in females (p < 0.001) but not in males (p = 0.617; Fig. 5). Trends from the gender analysis also held true when analyzing the number of AE per vaccination event, with no significant effects of either gender categories or gender scores (Fig. 5). Regardless of whether AE were conceptualized as a binary or continuous outcome, biological sex, but not gender, had a significant effect on AE.

### Sex steroid hormones and inflammatory cytokines contribute to the occurrence of adverse events in males

**Fig. 6:**
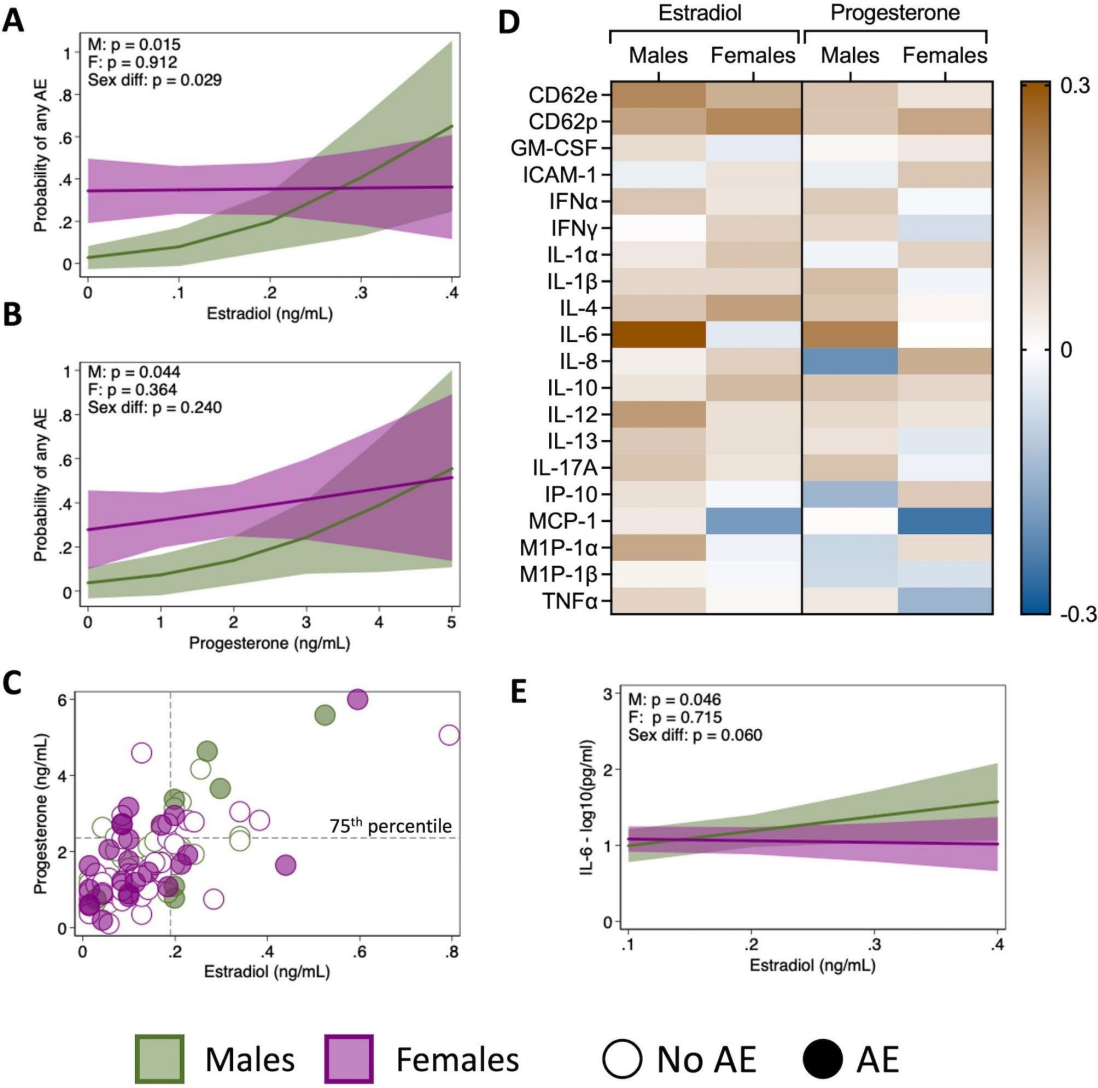
Relationship between sex steroid hormones, inflammatory cytokines, and adverse event (AE) reporting. The effect of estradiol (**A**) and progesterone (**B**) on the probability of reporting any AE was estimated for males and females using logistic regression models with interaction terms between the hormone and sex. The correlation between estradiol and progesterone concentrations is shown in **C**, where filled in circles represent participants who reported an AE, empty circles indicate participants who did not report an AE, and dashed lines indicate the 75th percentile of estradiol or progesterone concentrations. The correlations between estradiol or progesterone and a panel of twenty cytokines involved in inflammatory processes were assessed, with the strength of the correlation (r values) for males and females displayed (**D**). Cytokine concentrations were measured in picograms per milliliter, and values were log_10_-tranformed for analyses. The associations between estradiol and IL-6 concentration for males and female were tested using linear regression models with an interaction term between estradiol concentrations and sex (**E**)

We next sought to elucidate potential biological mechanisms underlying the observed sex difference in the occurrence of AE. Concentrations of estradiol, progesterone, testosterone, and cortisol were measured in plasma collected prior to vaccination in a subset of samples (‘serology subset’, Table 1). Only testosterone levels differed significantly by sex, with greater concentrations in older males than females (p < 0.001), and there were no effects of age or BMI on hormone concentrations (Supplemental Fig. 1). There was also no association between sex steroid hormones and gender categories (data not shown). Among males, there was a significant association between estradiol concentrations and the probability of reporting an AE (p = 0.015), but this effect was absent in females (p = 0.912), leading to a significant sex difference in the effect of estradiol on AE (p = 0.029, Fig. 6A). Similarly, progesterone concentrations were associated with the probability of reporting an AE in males (p = 0.044) but not in females (p = 0.364, Fig. 6B). Levels of estradiol and progesterone were correlated with each other in both males (r = 0.73) and females (r = 0.69). Interestingly, males who reported an AE tended to be in the highest quartile of both estradiol and progesterone, whereas females who reported AE had variable levels of both hormones (Fig. 6C). Neither testosterone nor cortisol had significant associations with AE for either males or females (Supplemental Fig. 2).

Based on literature suggesting a link between sex steroid hormones and cytokines in inflammatory pathways [18–23], we next investigated correlations between baseline levels of estradiol or progesterone and a panel of twenty cytokines associated with inflammation. The strongest correlation was observed between estradiol and IL-6 in males (r = 0.304; Fig. 6D). This correlation was present to a lesser extent with progesterone and entirely absent in females, potentially explaining why estradiol was not associated with the probability of reporting an AE in females. Further examining the relationship between IL-6 and estradiol using regression models revealed a significant association in males (p = 0.046) that was absent in females (p = 0.715), trending towards a significant sex difference in the effect of estradiol on IL-6 (p = 0.060, Fig. 6E). Together, these data point towards a mechanism through which higher levels of estradiol may contribute to a pro-inflammatory environment that results in AE in males and suggest that the biological processes leading the occurrence of AE post vaccination may be fundamentally different in males than in females.

## Discussion

In this longitudinal cohort study over four influenza seasons, biological sex was associated with the rate of AE following seasonal high dose influenza immunization in older adults to a greater extent than gender, as measured by the BSRI. Along with the profound and sex-specific effect of age on the probability of reporting an AE, these data support a biological, rather than a socio-cultural, mechanism for the higher rate of AE following immunization in females. We further found evidence to suggest that the processes that mediate AE following immunization may differ between males and females.

In males, estradiol was associated with both the inflammatory cytokine IL-6 and a higher probability of reporting an AE. Indeed, IL-6 has previously been associated with AE following receipt of the hepatitis B vaccine, although sex-specific effects were not investigated [27]. A sex difference in the relationship between hormones and inflammatory cytokines has previously been described in vitro, in which stimulation of peripheral blood mononuclear cells (PBMC) donated by males and females responded differently to increasing concentrations of exogenous estradiol [28]. Whereas male PBMC secreted increasing concentrations of TNFα and IL-6 in response to increasing estradiol stimulation, female PBMC did not secrete either cytokine, regardless of estradiol concentration [28]. The authors propose that the hypo-responsiveness of female PBMC to estradiol may be due to tolerance induced by exposure to high levels of estradiol during menstruation and pregnancy. We hypothesize that a similar mechanism may explain the association between baseline estradiol levels and AE in males that was absent in females.

We were unable to elucidate the pathways that contribute to AE in females with the available samples. This study was originally designed to assess immunogenicity of influenza vaccines, with samples collected at seven and 28 days post-vaccination [29]. The inflammatory mediators induced by vaccination that contribute to reactogenicity, however, peak at 24 h and return to baseline within three days [17]. We hypothesize that unlike in males, where AE appear to be mediated, in part, by baseline hormone and cytokine levels, AE may be mediated by the inflammatory response following vaccination in females. To capture this effect, future studies should collect samples within 24 h of vaccination to assess associations between sex steroid hormones, inflammatory cytokines, and AE. It is also important to note that the population consisted of post-menopausal females, with overall low levels of estradiol and progesterone. The effect of estradiol may be different in younger cohorts including pre-menopausal females. Furthermore, in addition to the immunological mechanisms that may contribute to the sex differences in AE that were explored here, there are also anatomical factors to consider [30, 31]. Females have smaller deltoid muscles than males, meaning that injections cause greater distension of the muscle, which may be associated with more pain [2, 14]. Females also have thicker subcutaneous skin layers, such that vaccines are more likely to be administered subcutaneously rather than intramuscularly, leading to more AE [14–16]. It is therefore likely that the biological cause of increased AE following immunization in females is multi-factorial and different from the mechanisms mediating AE in males.

Despite the well-documented link between gender and health outcomes, incorporation of robust measures of gender in biomedical research remains challenging [32]. Difficulties in measuring gender stem from its context-specific and fluid nature. Furthermore, gender refers to both identity (whether someone identifies as a man, woman, or gender minority) and power relations (inequitable access to resources, roles and practices, norms and values, and rules and decision-making) [33]. Here, we used a modified version of the short-form BSRI to measure gender. First developed in the 1970s [34], the tool has since been validated in pilot studies in Spanish and Brazilian older populations [35, 36] and found to be a practical and reliable instrument in a sample of nearly 2000 older adults from five international sites [37]. Gender roles measured by the 12-item BSRI have been used to study health outcomes, such as physical function and depression in older adults [38, 39]. The BSRI, however, has been criticized for relying solely on stereotypically masculine or feminine personality traits, which may be era- and culture-dependent and preclude considerations of gendered power relations [33, 40, 41]. Other gender scales, which aim to measure femininity, masculinity, and the power dynamics imbued in these roles, have been used across an array of medical fields, including cancer and heart disease [42–44]. Few of these tools are validated, however, and they are often context specific. It is thus possible that contributions of gender to AE reporting were not observed due the use of the BSRI but would have been captured using a different tool or form of measurement.

This study has several strengths and limitations. To our knowledge, this is the first study to comprehensively and quantitively measure both sex and gender in the context of adverse events following vaccination. This type of inter-disciplinary research is crucial to understanding the complex factors that lead to the occurrence and reporting of AE and ultimately the link between AE and vaccine uptake. Furthermore, use of data from a relatively large cohort over four influenza seasons resulted in a robust and well-powered dataset. In terms of limitations, rates of adverse events were measured by active self-report and may thus be biased by reporter subjectivity. This bias was limited by using active surveillance, meaning that participants were not expected to initiate contact with the study team but were called and asked about any vaccine reactions. It is possible, however, that what each participant considered to be an AE worthy of reporting affected analysis, particularly in terms of gender. In addition, there was no placebo group, therefore, we are unable to relate the AE directly to the influenza vaccine. Other placebo-controlled trials demonstrate that local AE, which we report as being greater for female than male participants, are less commonly associated with placebo than influenza vaccination [45], lending additional credibility to our findings. It also is important to note the lack of racial diversity in our cohort, which was predominantly Caucasian. Experiences and reporting of AE may differ by race, especially in the context of local AE, which can presently different in individuals who are Black, Indigenous, and People of Color [46].

## Conclusions

In conclusion, through an inter-disciplinary study of both the socio-cultural and biological factors that may contribute to the occurrence of AE following influenza vaccination in older adults, we identified a strong biological basis for a sex difference in the rate of AE. We further began to elucidate potential mechanisms for this sex difference and found evidence to suggest that the contributing biological pathways are fundamentally different in males and females. This work represents an important first step in understanding biological sex differences in vaccine safety outcomes and highlights the importance of reporting sex-disaggregated data. Ultimately, research on how vaccine outcomes differ by sex is crucial to developing tailored vaccine products or strategies that minimize the risk of AE and promote vaccine confidence.

## Methods

### Study population and protocol

Older adults were recruited from the Johns Hopkins Longitudinal Influenza Immunization Study of Aging over 75 years of age (JH LIISA 75+) cohort during the 2019-20, 2020-21, 2021-22, and/or 2022–2023 influenza seasons [29]. Individuals who had a history of allergic reaction to influenza vaccines or to eggs, were currently taking oral steroids, or had worsening or new-onset of immune-modulating conditions (e.g., rheumatoid arthritis, hematologic malignancies, etc.) were excluded. Sex and age were measured by self-report. After a pre-vaccination blood draw, participants received the high-dose inactivated influenza vaccine (Fluzone®High-Dose, Sanofi Pasteur, PA, USA).

### Adverse event reporting

Adverse events were solicited 6–8 days post-vaccination either over the phone or in-person. Participants were asked if they experienced the following local reactions (i.e., at the site of injection): redness, swelling, itching, warmth, pain on contact, and continuous pain. Participants were then asked if they experienced the following systemic reactions: redness, fever, sweating, chills, muscle aches, headaches, malaise, and insomnia. For each reported reaction, participants were asked the time of onset, the duration, and to rate the severity. Participants who reported at least one AE were then asked to describe the impact of the AE, in terms of ability to perform activities of daily living and treatment sought for the reaction.

### Gender measurement

Gender categories, femininity scores, and masculinity scores were derived from a modified version of the 12-item Bem Sex Role Inventory (BSRI), which has been used and validated in populations of older adults in multiple different cultures in the past decade [35–39]. The 12-item BSRI includes six instrumental/masculine (I/M) traits (leadership abilities, strong personality, acts as leader, dominant, makes decisions easily, defends own beliefs) and six expressive/feminine (E/F) traits (warm, gentle, affectionate, sympathetic, sensitive to other’s needs, tender)[35]. Following this scoring method, participants typically evaluate the extent to which a series of traits apply to them using a seven-point Likert scale [35]. To facilitate questionnaire administration over the phone during the COVID-19 pandemic, a five-point Likert scale was used. Each participant’s mean E/F and I/M scores were calculated and then compared to the sample median [35]. Those who were above the median on E/F traits but below on I/M traits were classified as feminine, while those above the median on I/M traits but below on E/F traits were classified as masculine. Individuals who were above or below the median on both E/F and I/M traits were classified as androgynous or undifferentiated, respectively.

### Microneutralization assay

Plasma samples were diluted with receptor destroying enzyme (RDE, Denka Seiken) at 1:3 ratio and incubated overnight at 37 °C followed by heat inactivation RDE at 56 °C for 35 min.

Samples were 2-fold serially diluted in infection media (1% Penicillin/Streptomycin, 1% Glutamax, 0.5% BSA, 5 µg/ml of N-acetyl-trypsin in Dulbecco’s Modified Eagle’s Medium), and mixed with 25 TCID50 of A/Guangdong-Maonan/SWL1536 (H1N1) vaccine strain or A/Hong Kong/ 2671/2019(H3N2) vaccine strain, and incubated at room temperature for 1 h.

The virus/serum mixture was transferred in duplicate into the 96-well cell culture plates containing confluent Madin-Darby Canine Kidney (MDCK) cells and incubated at 32 °C. After a 24 h incubation, plates were washed once with 1X PBS, fresh infection media was added, and cells were incubated for 7 days. Plates were fixed with 4% formaldehyde, stained with naphthol blue black solution. The neutralizing antibody titer was calculated as the highest plasma dilution that eliminated virus cytopathic effects in 50% of the wells.

### Hormone measurement

Concentrations of estradiol, progesterone, testosterone, and cortisol were measured in plasma samples collected prior to vaccination using a multiplex kit (MILLIPLEX® Multi-Species Hormone Magnetic Bead Panel, Millipore, catalogue #MSHMAG-21 K), as described by the manufacturer. Briefly, plasma samples were extracted using acetonitrile precipitation (ThermoScientific) and supernatants were dried using a Speed Vac machine. Assay buffer, 25µL of sample, standard, or control, HRP-conjugate, and antibody-immobilized beads were added to the plate and incubated at 4 °C while shaking for 16 h. Plates were then washed four times and 25µL of detection antibody was added to each well, followed by a one-hour incubation at room temperature with shaking. 25µL of Streptavidin-Phycoerythrin was then added to each well, and plates were incubated for 30 min at room temperature with shaking. Plates were then washed twice, and 100µL of curiox buffer (PBS with 1% BSA and 0.5% Tween 20) was added to each well. Plates were read on a MagPix™ Luminex instrument, and concentrations were calculated using a 5-parameter logistic curve-fitting method based on the mean fluorescence intensity.

### Cytokine Measurement

Concentrations of twenty cytokines involved in inflammatory responses were measured in plasma samples collected prior to vaccination using a multiplex kit (ProcartaPlex™ Multiplex Immunoassay, ThermoFisher Scientific, catalogue # EPX200-12185-901), as described by the manufacturer. Briefly, 50µL of the 20-plex bead mix was added to the plate and 25µL of sample, standard, or control were added to the designated wells and incubated at room temperature while shaking for 2 h. The plate was washed twice and 25µL of the detection antibody was added to the plate and incubated at room temperature with shaking for 1 h. The plate was then washed twice and 50µL of Streptavidin-PE was added and incubated at room temperature with shaking for 30 min, washed again twice, and resuspended in 120µL of wash buffer. Plates were read on a MagPix™ Luminex instrument. The mean fluorescence intensities were recorded and the concentrations for each cytokine were extracted using a 5-parameter logistic curve-fitting method using the ThermoFisher Scientific ProcartaPlex Analysis Software.

### Statistical analysis

Rates of each solicited AE were reported for males and females and were then summarized into four binary outcome measures for more complex analyses: whether the participant reported any AE, any local AE, any systemic AE, or any grade 2 AE. A grade 2 AE was defined as either having an impact on activities of daily living or requiring treatment [47]. Binary outcomes were then modelled using mixed-effects logistic regression models to account for repeat measurements on individuals who participated in multiple study years. As an alternate continuous outcome, the number of AE reported per vaccination event was modelled using mixed effects linear regression analysis. Base univariate models were first constructed to assess the association between sex, age, gender category, femininity, masculinity, and hormone levels. Multivariable regression models were then constructed using statistically significant predictors from the base models. Interactions between sex and aging were interrogated based on *a priori* hypotheses related to sex-specific effects of aging on vaccine outcomes [29, 48, 49]. Interaction models between hormone levels and sex were also used to investigate sex-specific biological processes. Correlations between hormones and cytokines were assessed using Pearson’s correlation coefficient. All p-values < 0.05 were considered statistically significant, and analyses were performed in Stata 15 (StataCorp).

### Electronic supplementary material

Below is the link to the electronic supplementary material.

Supplementary Material 1

## Data Availability

The datasets used and/or analyzed during the current study are available from the corresponding author on reasonable request.

## Abbreviations

AE: adverse events
BSRI: Bem Sex Role Inventory
IL-6: Interleukin 6
PBMC: peripheral blood mononuclear cells
