# Supplementary material for: The intersection of biological sex and gender in adverse events following seasonal influenza vaccination in older adults
Source: Immun Ageing. 2023 Aug 29;20:43. doi: 10.1186/s12979-023-00367-3 (PMC10463383; doi:10.1186/s12979-023-00367-3)
Supplement: Supplementary file 1 — Supplementary Material 1 [file 12979_2023_367_MOESM1_ESM.docx]

**The intersection of biological sex and gender in adverse events following seasonal influenza vaccination in older adults**

**Supplemental Materials**

**Table of contents**

[Supplemental Tables 2](#_Toc137643413)

[Supplemental table 1. Regression models investigating the effects of sex, the occurrence of adverse events, and their interaction on the neutralizing antibody response to vaccination 2](#_Toc137643414)

[Supplemental table 2. Regression models investigating the effects of sex, age, and their interaction on the odds of reporting an adverse event 3](#_Toc137643415)

[Supplemental table 3. The effects of gender categories and gender scores on the odds of reporting an adverse event 3](#_Toc137643416)

[Supplemental Figures 4](#_Toc137643417)

[Supplemental figure 1. Effect of sex and age on hormone levels. 4](#_Toc137643418)

[Supplemental figure 2. Impact of testosterone and cortisol on adverse event reporting. 5](#_Toc137643419)

# Supplemental Tables

## Supplemental table 1. Regression models investigating the effects of sex, the occurrence of adverse events, and their interaction on the neutralizing antibody response to vaccination

|  | **Males** | | | **Females** | | |  |
| --- | --- | --- | --- | --- | --- | --- | --- |
|  | **No AE** | **Any AE** | **p-value^a^** | **No AE** | **Any AE** | **p-value^a^** | **p-value^a^** |
| **N** | 39 | 7 |  | 42 | 24 |  |  |
| **GMT (CI)** |  |  |  |  |  |  |  |
| H1N1 D0 | 34.1 (24.6 - 47.3) | 59.4 (28.7 - 123.0) | 0.198 | 32.8 (24.9 - 43.2) | 38.9 (21.7 - 69.6) | 0.529 | 0.169 |
| H1N1 D28 | 174.9 (107.8 - 283.8) | 262.5 (88.0 - 782.8) | 0.443 | 137.9 (98.6 - 192.9) | 190.3 (110.8 - 326.7) | 0.330 | 0.223 |
| H3N2 D0 | 40.7 (24.0 - 69.0) | 22.1 (9.3 - 52.3) | 0.339 | 48.8 (30.4 - 78.2) | 42.4 (21.4 - 84.0) | 0.725 | 0.344 |
| H3N2 D28 | 338.0 (195.7 - 583.7) | 195.0 (18.9 - 2009.5) | 0.429 | 304.5 (186.2 - 498.1) | 349.0 (175.6 - 693.5) | 0.753 | 0.710 |
| **GMFR (CI)** |  |  |  |  |  |  |  |
| H1N1 | 5.1 (3.6 - 7.3) | 4.4 (1.3 - 14.6) | 0.748 | 4.2 (3.0 - 5.9) | 4.9 (2.9 - 8.4) | 0.600 | 0.912 |
| H3N2 | 8.3 (5.7 - 12.1) | 8.8 (1.5 - 51.0) | 0.900 | 6.2 (4.3 - 9.0) | 8.2 (5.1 - 13.3) | 0.372 | 0.487 |
| **Seroconversion**  **- n (%)** |  |  |  |  |  |  |  |
| H1N1 | 26 (66.7) | 3 (42.9) | 0.241 | 27 (64.3) | 19 (79.2) | 0.211 | 0.241 |
| H3N2 | 32 (82.1) | 5 (71.4) | 0.519 | 31 (73.8) | 20 (83.3) | 0.378 | 0.519 |

^a^ P-values indicate the significance of the difference between those who had an AE and those who did not among males, females, or the overall population. P-values were derived from linear (GMT and GMFR) or logistic (seroconversion) regression models with binary main effects for sex and the presence of an AE and an interaction term between the main effects.

Abbreviations: CI: confidence interval; D: day; Diff: difference; GMT: geometric mean titer; GMFR: geometric mean fold rise

## Supplemental table 2. Regression models investigating the effects of sex, age, and their interaction on the odds of reporting an adverse event

| **OR (p-value)** | **Any AE** | **Local AE** | **Systemic AE** | **Grade 2 AE** |
| --- | --- | --- | --- | --- |
| **Base models** |  |  |  |  |
| Female sex | 4.038 (0.0007) | 5.164 (0.0002) | 2.870 (0.1354) | 3.7509 (0.0623) |
| Older age | 0.849 (0.0001) | 0.840 (0.0001) | 0.805 (0.0069) | 0.9377 (0.2703) |
| **Adjusted models** |  |  |  |  |
| Female sex | 3.373 (0.0024) | 4.343 (0.0006) | 1.948 (0.3229) | 3.4318 (0.0790) |
| Older age | 0.864 (0.0002) | 0.857 (0.0002) | 0.819 (0.0106) | 0.9525 (0.4012) |
| **Interaction model** |  |  |  |  |
| Older age in males | 0.941 (0.3479) | 0.916 (0.2264) | 1.026 (0.7976) | 1.0491 (0.6705) |
| Older age in females | 0.830 (0.0002) | 0.834 (0.0003) | 0.737 (0.0021) | 0.9256 (0.2400) |

## Supplemental table 3. The effects of gender categories and gender scores on the odds of reporting an adverse event

|  | **All** | **Male** | **Female** |
| --- | --- | --- | --- |
| **Descriptive analysis - Any AE/N (%)** |  |  |  |
| Masculine | 20/65 (31) | 8/37 (22) | 12/28 (43) |
| Feminine | 17/70 (24) | 4/21 (19) | 13/49 (27) |
| Androgynous | 28/115 (24) | 4/35 (11) | 24/80 (30) |
| Undifferentiated | 18/89 (20) | 5/34 (15) | 13/55 (24) |
| **Regression analysis - OR (p-value)^a^** | | | |
| **Gender roles^b^** |  |  |  |
| Masculine | 1.491 (0.430) | 1.333 (0.735) | 2.065 (0.263) |
| Androgynous | 1.009 (0.985) | 0.492 (0.439) | 1.266 (0.641) |
| Undifferentiated | 0.818 (0.676) | 0.775 (0.773) | 0.888 (0.832) |
| **Gender scores** |  |  |  |
| Femininity | 0.785 (0.392) | 0.578 (0.283) | 0.739 (0.384) |
| Masculinity | 1.092 (0.669) | 0.750 (0.474) | 1.255 (0.338) |

^a^ Outcome = any AE

^b^ Reference group = feminine

# Supplemental Figures

## Supplemental figure 1. Effect of sex, age, and BMI on hormone levels.

Hormones were measured in plasma samples collected prior to influenza vaccination during the 2020-2021 season. The concentrations of estradiol (**A**), progesterone (**B**), testosterone (**C**), and cortisol (**D**) were compared between males and females using simple t-tests. The associations between estradiol (**E**), progesterone (**F**), testosterone (**G**), or cortisol (**H**) and age were tested for males and females using linear regression models with an interaction term between age and sex. Similarly, the associations between estradiol (**I**), progesterone (**J**), testosterone (**K**), or cortisol (**L**) and BMI were tested for males and females using linear regression models with an interaction term between BMI and sex.


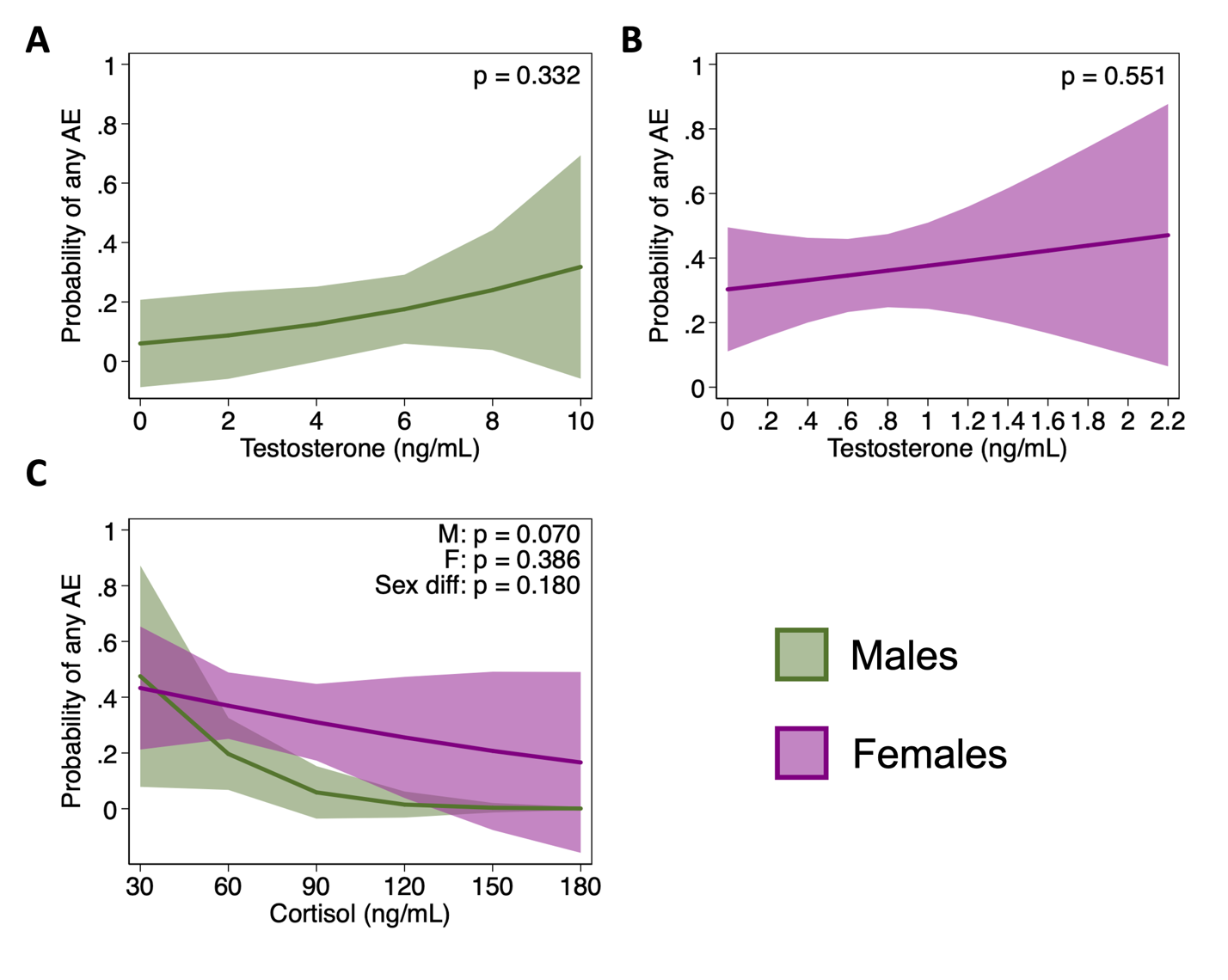


## Supplemental figure 2. Impact of testosterone and cortisol on adverse event reporting.

The effect of testosterone (**A-B**) and cortisol (**B**) on the probability of reporting any AE was estimated for males and females using logistic regression models with interaction terms between hormone concentrations and sex.
